# When Infection Meets Inflammation: *Listeria monocytogenes* and Host Signaling Pathways

**DOI:** 10.3390/biology15070541

**Published:** 2026-03-27

**Authors:** Yanyan Jia, Ke Yang, Rongxian Guo, Ke Ding, Shaohui Wang, Songbiao Chen

**Affiliations:** 1 Laboratory of Functional Microbiology and Animal Health, College of Animal Science and Technology, Henan University of Science and Technology, Luoyang 471023, China; 2 Shanghai Veterinary Research Institute, Chinese Academy of Agricultural Sciences, Shanghai 200241, China; 3 Ministry of Education Key Laboratory for Animal Pathogens and Biosafety, Zhengzhou 450000, China; 4 College of Animal Science and Technology, Henan Institute of Science and Technology, Xinxiang 453003, China

**Keywords:** *L. monocytogenes*, pathogenesis, inflammasome, activation, molecular mechanism

## Abstract

*Listeria monocytogenes* (*L. monocytogenes*) is a significant zoonotic pathogen responsible for listeriosis, a foodborne infection with high mortality. The inflammasome, an innate immune complex, plays a critical role in controlling pathogenic infections through its rapid inflammatory output. During *L. monocytogenes* infection, pore-forming toxins such as listeriolysin-O and flagellin are quickly recognized by pattern recognition receptors (PRRs), triggering inflammatory responses and activating the host’s anti-infection immunity. However, excessive or chronic inflammasome activation and subsequent interleukin-1β (IL-1β) release are implicated in the pathogenesis of *L. monocytogenes*. Although inflammasome activation is an effective defense against *L. monocytogenes*, the bacterium has evolved multiple mechanisms to inhibit this immune pathway. Hence, research on inflammasomes activation is crucial for better understanding the pathogenic mechanism of *L. monocytogenes*. In this review, we highlight recent advances in the understanding of the molecular mechanisms of inflammasome activation by *L. monocytogenes* infection. We then discuss advances in the role of the inflammasome pathway in the pathogenesis of *L. monocytogenes*, along with an overview of the applications of inflammasome inhibitors. Extensive studies into the mechanisms by which *L. monocytogenes* activates the inflammasome could lead to the discovery of novel therapeutic targets and strategies to fight *L. monocytogenes* infections.

## Simple Summary

*Listeria monocytogenes* is a foodborne bacterium that can cause serious and sometimes fatal infections. The host employs a defense mechanism called the inflammasome, which triggers inflammation when it detects harmful bacterial components helping to fight infection. However, an overactive inflammasome can release excessive interleukin-1β, causing tissue damage and worsening disease. Thus, while essential for controlling bacterial growth, it can also contribute to harm if not regulated. *L. monocytogenes* has evolved strategies to evade this immune response. Understanding how the bacteria and inflammasome interact is crucial for uncovering infection mechanisms and developing targeted therapies. This review explains inflammasome activation during Listeria infection, highlights its dual protective and damaging roles, and discusses potential therapies aimed at modulating inflammasome activity.

## 1. Introduction

*L. monocytogenes* is a Gram-positive facultative anaerobic pathogen with strong tolerance to external environmental conditions and is widely present in the environment [1], causing infection in humans and various animals. Under the influence of various virulence factors, *L. monocytogenes* can cross the intestinal barrier, placental barrier, and blood–brain barrier, which allows the bacteria to cause disorders such as sepsis, meningitis, and miscarriages in both humans and animals, with a mortality rate of up to 30% [2]. Epidemiological data indicate that the incidence of listeriosis varies markedly across regions, with the highest reported incidence observed in European countries. For example, a study from northern Spain reported an average annual incidence of 1.55 cases per 100,000 population for non-pregnancy-associated cases [3], whereas an estimated incidence of 0.77 cases per 100,000 population was reported for central Italy using a capture–recapture approach [4]. In Central European countries such as the Czech Republic, the reported incidence was 0.33 cases per 100,000 population, with regional variation reaching as high as 0.6 cases per 100,000 [5]. These figures are clearly higher than the EU average incidence, highlighting the substantial disease burden borne by European populations. However, the pathogenicity of *L. monocytogenes* remains unclear. Therefore, by clarifying the pathogenicity of monocytic proliferative *Listeria*, new adjuvant therapeutic techniques can be discovered to prevent and control listeriosis.

The inflammasome is an innate immune complex that plays a critical role in controlling pathogenic infections through rapid inflammatory responses. Its activation is essential for effective host immunity against both pathogens and sterile insults; however, excessive activation can be detrimental to the host [6]. To date, *L. monocytogenes* can activate a variety of inflammasomes via its pore-forming toxin listeriolysin-O, flagellin, or DNA released through bacteriolysis, which are recognized by a distinct pattern of recognition receptors (PRRs) including NOD-like receptors, RIG-I-like receptors, and AIM2-like receptors [7,8]. By self-oligomerization, these receptors create inflammasomes that mediate pyroptosis and release proinflammatory cytokines, including IL-1β and IL-18, which initiate a series of inflammatory immunological responses [9]. Hence, inflammasomes are essential to the process by which *L. monocytogenes* survives and multiplies within host cells after infection, leading to a strong immunological response and pathological consequences in the host. Thus, a better understanding of the relationship between *L. monocytogenes* and inflammasomes not only offers novel insights into the prevention of listeriosis but also promotes the sustainable growth of animal husbandry and protects the health of the public.

## 2. Overview of *L. monocytogenes*

*L. monocytogenes* is an intracellular pathogenic bacterium that invades macrophages and non-phagocytic cells, which infect humans and animals and causes listeriosis characterized by a high mortality rate (up to 30%). The ability to survive unfavorable conditions determines the ubiquitous nature of *L. monocytogenes* in the environment. High virulence and ubiquitous nature of *L. monocytogenes* may pose a relevant public health problem because of some genomic and pathogenicity islands.

### 2.1. Pathogenicity of L. monocytogenes 

**Figure 1:**
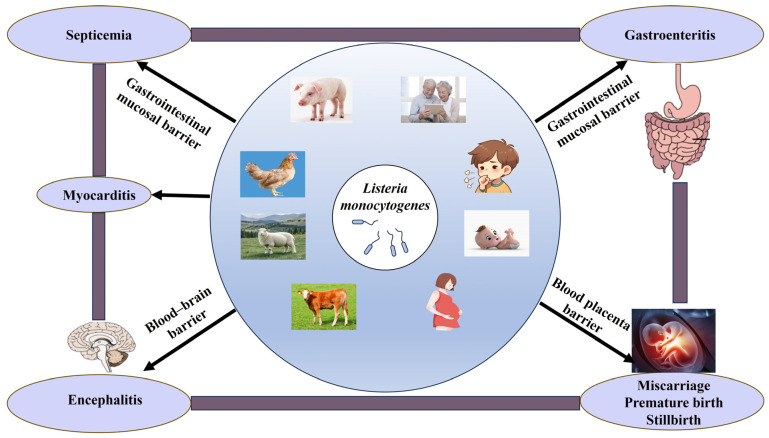
The hazards of *L. monocytogenes*.

*L. monocytogenes* is a food-borne pathogen that can penetrate the intestinal barrier and spread into the bloodstream and lymphatic system, subsequently reaching the liver and spleen for replication. Animals infected with *L. monocytogenes* can have sepsis, encephalitis, meningitis, miscarriage, stillbirth, and gastroenteritis as their main symptoms (Figure 1) [10]. *L. monocytogenes* can cross the placental and blood–brain barriers in immunocompromised people, especially elderly and pregnant women, leading to neurological listeriosis and maternal–fetal listeriosis (Figure 1) [11]. Although the incidence of listeriosis is relatively low [12], the mortality rate is quite high [13], posing a significant threat to livestock production.

*L. monocytogenes* is a zoonotic pathogen, which can infect various animals and especially elderly and pregnant women. Hosts infected with *L. monocytogenes* can experience septicemia, gastroenteritis, myocarditis, encephalitis, miscarriage, and stillbirth, as it can cross the gastrointestinal mucosal, placental and blood–brain barriers.

### 2.2. Infectious Life Cycle of L. monocytogenes

**Figure 2:**
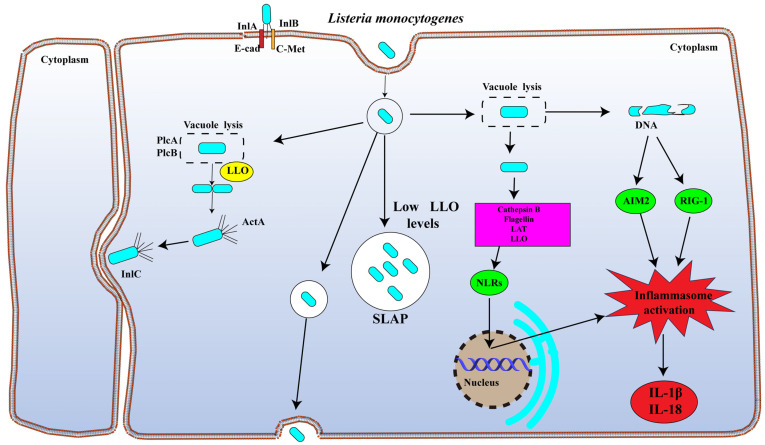
Intracellular life history of *L. monocytogenes* infection.

*L. monocytogenes* is an intracellular parasite, and its pathogenicity is associated with various virulence genes. Bacteria are phagocytosed by epithelial cells to transmigrate across the mucosal barrier, where it enters the bloodstream and disseminates throughout the body. The nervous system and placenta are the most affected infection sites. The infection cycle of *L. monocytogenes* mainly consists of three stages, invasion and adhesion to host cells, intracellular proliferation within host cells, and intercellular dissemination, all of which require the involvement of specific factors [14]. The coordinated action of particular virulence factors, including listeriolysin O (LLO), internalins (InlA and InlB), actin assembly inducing protein (ActA), and phospholipases (PlcA and PlcB), is necessary for the successful completion of each stage [15]. One of the main virulence factors of *L. monocytogenes* is LLO, a pore-forming toxin that is encoded by the *hly* gene. It disrupts the phagocytic vacuole membrane, allowing bacteria to enter the cytoplasm and proliferate. This process is essential for lysis and escape from the phagosome [16]. InlA is essential for *L. monocytogenes* to pass through the intestinal epithelium and placental barrier [17], whereas InlB allows *L. monocytogenes* to evade immune monitoring and infect liver cells. ActA mediates actin polymerization-driven bacterial motility, promoting the spread of *L. monocytogenes* between cells and within host tissues [18]. PlcA facilitates the escape of the pathogen from the phagosome, whereas PlcB aids in the evasion of the host’s autophagic defences (Figure 2) [2,19].

The innate immune system is activated first after *L. monocytogenes* infection. The host’s initial line of defence against pathogens is the innate immune response, which uses germline-encoded pattern recognition receptors to identify bacteria and viruses. A key function of the innate immune system is the activation of inflammasomes [20], which can mediate the activation of caspase-1, leading to the secretion of the proinflammatory cytokines IL-1β and IL-18 and initiating the process of “pyroptosis” [21]. Activation of the inflammasome induced by *L. monocytogenes* infection exacerbates the host’s pathological condition.

*L. monocytogenes* is engulfed by phagocytic cells or uptaken through binding E-cad and C-Met in non-phagocytic cells. It can transcytose across the cell within a vacuole in goblet cells or replicate in spacious *Listeria*-containing phagosomes (SLAPs) in some macrophages. Some bacteria escape from the vacuole by the action of virulence factor Listeriolysin O (LLO), phospholipase A (PlcA) and PlcB. Upon vacuolar escape, *L. monocytogenes* subsequently polymerizes actin and can spread from cell to cell. During infection, NLRs, AIM2 and RIG-1 inflammasomes are activated by *L. monocytogenes* PAMPs, which result in the production of IL-1β and IL-18 and mount an antibacterial response. NLRs, NOD-like Receptors, AIM2, Absent in melanoma 2, RIG-1, Retinoic acid-inducible gene-1, SLAP, spacious Listeria-containing phagosome, E-cad, E-cadherin, C-Met, hepatocyte growth factor receptor, PAMPs, pathogen-associated molecular patterns. (InlA: Internalin A; InlB: Internalin B; InlC: Internalin C; PlcA/PlcB: Phosphatidylinositol-specific phospholipase C; ActA: Actin assembly-inducing protein; E-cad: E-cadherin; c-Met: Hepatocyte growth factor receptor; SLAP: Spacious Listeria-containing vacuoles; LTA: Lipoteichoic acid; LLO: Listeriolysin O; NLRs: NOD-like receptors; IL-1β: Interleukin-1 beta; IL-18: Interleukin-18; AIM2: Absent in melanoma 2; RIG-1: Retinoic acid-inducible gene-1).

## 3. The Role and Function of Inflammation in Pathogen Infections

Inflammasomes play crucial roles in the innate immune response, forming multiprotein complexes in response to various physiological and pathogenic stimuli [22]. Typically, an inflammasome consists of three components: a sensor molecule, the adapter ASC, and the effector caspase-1 [23]. The N-terminus of the sensor molecule contains either a CARD domain or a PYD domain. ASC is composed of a PYD and a CARD, and caspase-1 also has a CARD [24]. Both PYD and CARD can cause oligomerization. The ASC protein promotes the binding of receptor proteins and adaptor proteins via CARD-CARD or PYD-PYD interactions, allowing the inflammasome complex to be assembled and activated [25]. Multiple inflammasome types have been identified, and each inflammasome is associated with a distinct pattern of recognition receptors (PRRs). These receptors can identify different damage-associated molecular patterns (DAMPs) or pathogen-associated molecular patterns (PAMPs) and react appropriately. The PRR family includes NOD-like receptors, RIG-I-like receptors, and AIM2-like receptors (Table 1).

Inflammasome activation is an important innate immune defense mechanism against bacterial infection; conversely, bacteria express virulence determinants that counteract inflammasome activation. Bacteria encode multiple effectors to directly inhibit components of the inflammasome pathway, from inhibition of sensing mechanisms to inhibition of GSDMD pore formation. *Pseudomonas aeruginosa* can cause both acute and chronic infections. The secretory structural protein VgrG2b from *P. aeruginosa* is recognized and cleaved by caspase-11, releasing a free C-terminal fragment. This VgrG2b C-terminus binds to NLRP3, inhibiting NLRP3 inflammasome activation by blocking the interaction between NEK7 and NLRP3 [14]. Taken together, inflammation is a double-edged sword. Dysregulation of pro-inflammatory or anti-inflammatory responses may result in pathological complications—excessive inflammation causing tissue damage, or uncontrolled fibrosis due to prolonged anti-inflammatory signaling.

## 4. Activation of Inflammasomes Induced by *L. monocytogenes* Infection

### 4.1. NOD-like Receptors

NOD-like receptor proteins are intracellular pattern recognition receptors, and 22 human NLR family proteins and 34 mouse NLR family proteins have been identified [43].

These receptors are generally classified into five subfamilies based on their N-terminal structures: NLRA, NLRB, NLRC, NLRP, and NLRX. Among these, NLRP3, NLRC4, NLRC5, NLRP1, and NLRC5 are the most extensively studied.

#### 4.1.1. NLRP3 Inflammasome

The NLRP3 inflammasome is the most extensively studied member of the NOD-like receptor family and is capable of activating caspase-1 and inducing inflammation in response to both endogenous and exogenous stimuli [44,45]. The NLRP3 inflammasome is activated by a variety of agonists, including PAMPs and DAMPs, and its activation mechanism is quite complex [46]. The activation of the typical NLRP3 inflammasome requires two steps: in the priming phase, TLRs and cytokine receptors that identify PAMPs or DAMPs upregulate NLRP3 and IL-1β transcription. Subsequently, PAMPs and DAMPs facilitate the assembly of the NLRP3 inflammasome, leading to the maturation and release of inflammasome-mediated cytokines via caspase-1 [47]. The activation phase includes several critical processes: (1) Ion flux. The efflux of K^+^, efflux of Cl^−^, influx of Na^+^, and mobilization of Ca^2+^ are essential for NLRP3 activation [26,27]. (2) Lysosomal rupture by a series of microparticles [27]. (3) NLRP3 inflammasome activation is closely associated with ROS; inhibiting ROS suppresses the activation of the NLRP3–caspase 1–IL 1β pathway [28]. (4) Release of mitochondrial DNA [27]. (5) A serine/threonine kinase involved in mitosis, NEK7 [29]. Notably, the activation of the NLRP3 inflammasome does not always adhere to the two-step activation model. During infections with Gram-negative bacteria, intracellular lipopolysaccharides (LPSs) can be sensed by human caspase-4, caspase-5, and mouse caspase-11, leading to atypical NLRP3 inflammasome activation [30].

**Figure 3:**
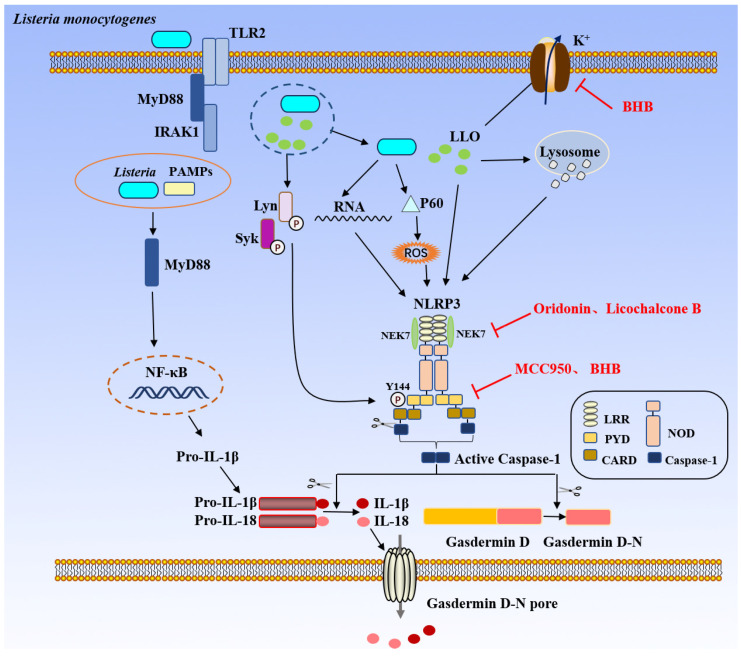
NLRP3-mediated activation of the inflammasome by *L. monocytogenes*.

*L. monocytogenes* enters the phagosomes of infected cells and lyses the phagosomal membrane with its released LLO, which spares the bacteria from death by allowing them to escape the acidic environment. Inflammasomes are triggered by germs that break free into the cytoplasm, with the NLRP3 inflammasome being activated initially [48]. Infection with *L. monocytogenes* can trigger the formation of the NLRP3 inflammasome in trophoblasts and RAW264.7 macrophages and activate the NLRP3 inflammasome in microglia through the NF-κB pathway [49,50,51]. The mechanism by which *L. monocytogenes* activates the NLRP3 inflammasome is relatively complex and involves multiple components. *L. monocytogenes* triggers NLRP3 inflammasome activation in an ASC-dependent manner [52], which is primarily associated with LLO [53]. Following *L. monocytogenes* infection of LLO-deficient B6 cells, there was a significant decrease in the release of IL-1β, caspase-1 maturation, and activation of apoptosis. Subsequent tests revealed that IL-1β release was significantly reduced, indicating the critical role of LLO in stimulating the formation of the NLRP3 inflammasome in macrophages during *L. monocytogenes* infection. Further studies indicated that LLO-induced NLRP3 inflammasome activation plays a key role in *L. monocytogenes* induced pregnancy failure. How does LLO activate the NLRP3 inflammasome? *L. monocytogenes* may internalize and then quickly rupture the phagosomal membrane via the virulence factor LLO, which allows the germs to escape into the cytoplasm. As a result, phagosomal disruption-induced events may cause NLRP3 activation [54]. When *L. monocytogenes* infects human peripheral blood monolayer cells (PBMCs), the bacteria secreted LLO that damages phagosome or lysosome membranes and releases cathepsin B. Both the release of cathepsin B and the rupture of the phagosome caused by LLO are detectable by NLRP3 [55]. Extracellular LLO can increase cell membrane permeability [56], causing potassium (K^+^) to efflux through the cell membrane, which in turn induces NLRP3 activation. Notably, the activation of NLRP3 by extracellular LLO is independent of cathepsin B release and is solely related to K^+^ efflux [57]. The activation of the NLRP3 inflammasome is induced by the threonine residue T223 in domain 3 of LLO, and this mechanism is independent of its pore-forming activity. The amino acid residue T223 of LLO is necessary for optimal interaction between LLO and lipid rafts, facilitating the phosphorylation of ASC at the Y144 residue through Lyn-Syk signaling, thus triggering the activation of the Lyn-Syk signaling cascade [58]. In addition to LLO, the NLRP3 inflammasome can also be triggered by the RNA of *L. monocytogenes* and the released virulence component P60 [8]. Research has demonstrated that the P60 protein secreted by *L. monocytogenes* can cause significant reactive oxygen species production in host cells, which in turn triggers the NLRP3 inflammasome [57]. The activation of the inflammasome involves the transcriptional upregulation of NLRP3, which is mediated by the expression of IL-1β, IL-18, and the inactive form of caspase-1 through the NF-κB pathway, as well as the ubiquitination and phosphorylation of ASC [59]. Both NLRP3 and pro-IL-1β are elevated because the NF-κB transcriptional pathway is activated [60]. TLR2 identifies *L. monocytogenes* lipoproteins on the cell surface during the activation phase [48]. Early in the course of infection, *L. monocytogenes* can quickly activate the NLRP3 inflammasome as long as the TLR pathway is intact [8]. Most TLRs, IL-1β, and IL-18 receptors receive downstream signals via the adapter molecule MyD88 [48]. Interleukin-1 receptor-associated kinase 1 (IRAK1) is a crucial mediator in the fast activation process of the inflammasome and is necessary for the inflammasome-dependent early warning response to pathogen infection. The MyD88-IRAK4-IRAK1 pathway can activate the NLRP3 inflammasome in macrophages infected with *L. monocytogenes* in as little as 60 min [7]. The activation of MST1/2 and ALK mediates this induced inflammatory response. Specifically, MST1/2-ALK promotes NEK7 binding to NLRP3 through JNK, mediating the activation of the NLRP3 inflammasome and increasing the maturation and release of the proinflammatory cytokine IL-1β (Figure 3) [61]. NLRP3 inflammasome activation produces active caspase-1, whcih cleaves pro-IL-1β and pro-IL-18 and gasdermin D to produce active gasdermin D-N. Gasdermin D-N forms pores, leading to membrane rupture, and IL-1β and IL-18 are secreted outside the cell through these pores. IL-1β and IL-18 not only amplify local inflammatory responses, but also play important roles in adaptive immunity. For instance, IL-1β is involved in the differentiation of naïve CD4^+^ T cells into inflammation-associated subsets, whereas IL-18 contributes to the promotion of Th1-related immune responses. In addition, inflammasome signaling can influence the maturation and functional polarization of antigen-presenting cells and other myeloid populations, thereby further shaping the broader immune microenvironment. These data indicate that some virulence factor from *L. monocytogenes* can activate NLRP3 inflammasome, which play a crucial role in the pathogenesis of *L. monocytogenes*.

Upon infection, *L. monocytogenes* PAMPs are sensed on the surface of cells by Toll-like receptors (TLRs), which sense bacteria in the phagosome, leading to nuclear factor-κB (NF-κB) activation and the upregulation of pro-IL-1β transcription. Some bacteria escape from the vacuole by the action of virulence factor Listeriolysin O (LLO), phospholipase A (PlcA) and PlcB. NLRP3 inflammasome is activated by *L. monocytogenes* PAMPs (LLO, its own RNA and P60 protein) in the cytoplasm. Such as LLO activates NLRP3 inflammasome by causing the release of cathepsin B and K^+^ efflux through lysosomal rupture and promoting the phosphorylation of ASC Y144 through the Lyn-Sky pathway. NLRP3 inflammasome activation produces active caspase-1, whcih cleaves pro-IL-1β and pro-IL-18 and Gasdermin D to produce active Gasdermin D-N. Gasdermin D-N forms pores, leading to membrane rupture, and IL-1β and IL-18 are secreted outside the cell through these pores. After the addition of inhibitors, MCC950 and BHB effectively suppress ASC oligomerization. Oridonin and licochalcone B exert their inhibitory effects by disrupting the interaction between NLRP3 and NEK7. In addition, BHB can block potassium efflux, thereby inhibiting activation of the NLRP3 inflammasome. (TLR2: Toll-like receptor 2; MyD88: Myeloid differentiation primary response 88; IRAK1: Interleukin-1 receptor–associated kinase 1; PAMPs: Pathogen-associated molecular patterns; NF-κB: Nuclear factor-κB; LLO: listeriolysin O; Lyn: Lyn proto-oncogene, Src family tyrosine kinase; Syk: spleen tyrosine kinase; ROS: Reactive Oxygen Species; NEK7: NIMA-related kinase 7; Pro-IL-1β: Pro-Interleukin-1 beta; Pro-IL-18: Pro-Interleukin-18; IL-1β: Interleukin-1 beta; IL-18: Interleukin-18; LRR: Leucine-Rich Repeat(s); PYD: Pyrin domain; CARD: Caspase Activation and Recruitment Domain; NOD: Nucleotide-binding and Oligomerization Domain; Caspase-1: Cysteine-dependent aspartate-directed protease-1; NLRP3: NOD-like receptor family, pyrin domain containing 3; BHB: Beta-hydroxybutyrate).

#### 4.1.2. NLRC4 Inflammasome

NLRC4 is a cytoplasmic member of the NOD-like receptor family. The NLRC4 inflammasome can detect two flagellar proteins and type III secretion system (T3SS) components, the rod and needle proteins, which act as ligands for NLR family apoptosis-inhibiting proteins (NAIPs). Both flagellins from *Legionella pneumophila*, a Gram-negative bacterium, and *Salmonella Typhimurium* can activate NLRC4, which in turn activates caspase-1. The specificity of the NLRC4 inflammasome is dictated by NAIP; NAIP1 interacts with the T3SS needle protein, NAIP2 works as the T3SS rod protein’s particular receptor [31], and NAIP5 and NAIP6 react exclusively to bacterial flagellin [62]. Nevertheless, human NLRC4 inflammasome only recognizes the T3SS needle protein, and there is only one family member in humans, hNAIP. In mouse and human macrophages, NLRC4 inflammasome can be activated by the T3SS needle protein [63]. Furthermore, serine 533 phosphorylation is thought to be essential for NLRC4 function. Procaspase-1 cannot be recruited, and inflammasome specks cannot be assembled by the phosphorylation-deficient NLRC4 mutant S533A. The activation of the NLRC4 inflammasome and the induction of conformational changes required for host innate immunity may be caused by the phosphorylation of NLRC4 at S533 [64].

**Figure 4:**
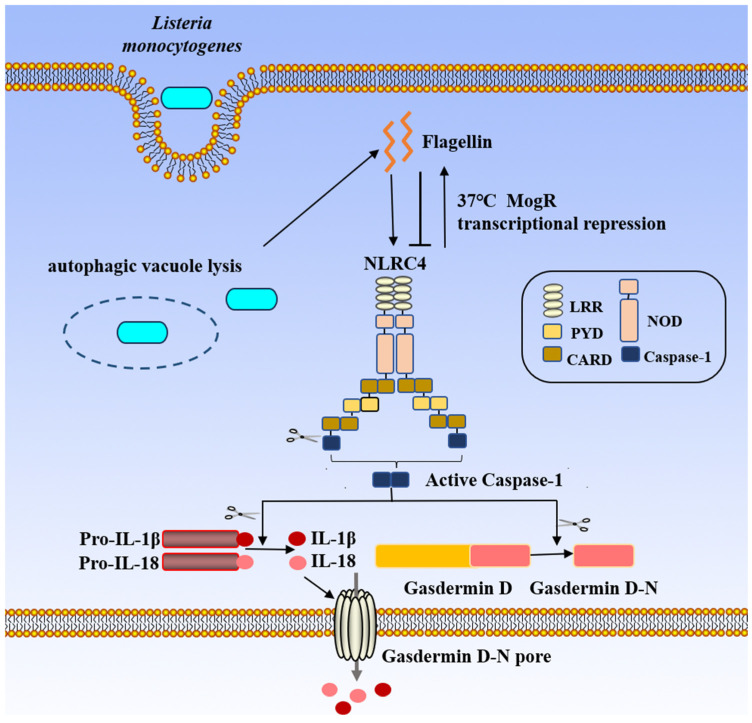
NLRC4-mediated activation of the inflammasome by *L. monocytogenes*.

In NLRC4^−/−^ cells infected with *L. monocytogenes*, the levels of caspase-1 activation and IL-1β secretion are reduced [57]. Furthermore, a recombinant strain of *L. monocytogenes* was constructed to express *Legionella pneumophila* flagellin ectopically by linking the flagellin protein to the actA regulatory element, allowing expression of the flagellin protein after it entered the cytoplasm. Compared with the wild-type *L. monocytogenes* strain, the recombinant strain that secretes flagellin specifically activates the NLRC4 inflammasome, leading to increased IL-1β secretion and promoting host cell death [65,66]. However, virulence of *Listeria* mutants expressing *Legionella pneumophila* flagellin is lower than that of wild-type LMs in bone marrow-derived macrophages and intravenous injection-infected mice. This decrease in virulence in vivo is attributed to the suppressed expression of the NLRC4 inflammasome [7]. The specific reason is that temperature plays an important role in the expression level of flagella in *L. monocytogenes*. At 22~25 °C, the bacteria may create four flagella; however, only one flagellum forms at 32 °C, suggesting that flagellum expression is repressed. In vivo, *L. monocytogenes* may use the transcriptional repressor MogR to restrict the expression of its flagellin protein under physiological conditions at 37 °C, which would prevent NLRC4 activation and reduce the recombinant strain’s pathogenicity (Figure 4) [8]. The results indicate that flagellin by activating NLRC4 participates in the pathogenesis of *L. monocytogenes*.

*L. monocytogenes* escape from the vacuole by the action of virulence factor Listeriolysin O (LLO), phospholipase A (PlcA) and PlcB. Listeria flagellin is recognized by NLRC4 inflammasome sensor localized in the cytosol to trigger inflammasome activation. But NLRC4 activation is inhibited because MogR (Motility Regulator G) inhibits the expression of flagellin at 37 °C. NLRC4 inflammasome activation cause the production and release of IL-1β and IL-18 and cell pyroptosis. (Pro-IL-1β: Pro-Interleukin-1 beta; Pro-IL-18: Pro-Interleukin-18; IL-1β: Interleukin-1 beta; IL-18: Interleukin-18; LRR: Leucine-Rich Repeat(s); PYD: Pyrin domain; CARD: Caspase Activation and Recruitment Domain; NOD: Nucleotide-binding and Oligomerization Domain; Caspase-1: Cysteine-dependent aspartate-directed protease-1; NLRC4: NOD-like receptor family, CARD domain containing 4).

#### 4.1.3. NLRC5 Inflammasome

NLRC5, the largest member of the NOD-like receptor family, is expressed primarily in the bone marrow and lymphoid cells and plays a crucial role in innate immune signaling and antiviral immune responses [67,68]. NLRC5 activates the NF-κB pathway in LPS-stimulated microglia, regulating the production of proinflammatory cytokines. Reducing NLRC5 expression inhibits the release of proinflammatory cytokines (IL-1β, IL-6, and TNF-α) in LPS-induced microglia and cell lines [36]. The activation of NLRC5 by LPS is inhibited by the lack of STAT1, and the expression of NLRC5 caused by LPS is dependent on STAT1 signaling [68]. Studies have demonstrated that NLRC5 inhibits NF-κB activation by interacting with IKKα and IKKβ and interacts with RIG-I through its CARD domain to suppress its signaling, thereby weakening RLR-mediated innate antiviral immune responses. However, some studies have indicated that NLRC5 strengthens RLR-mediated antiviral innate immune responses by increasing the activation of IFN-β and the NF-κB promoter. This finding implies that RLR-mediated signaling pathways can be bidirectionally regulated by NLRC5 [69]. Furthermore, the activation of NLRP3-mediated inflammasomes is also partially impaired in NLRC5-deficient mice, suggesting a shared activation process between NLRC5 and NLRP3 that eventually results in NLRP3 activation and promotes the formation of inflammasomes. While NLRC5 binds to NLRP3 via its NACHT domain, it is still unclear how NLRC5 promotes NLRP3 inflammasome activation [70].

When *L. monocytogenes* is injected into NLRC5^−/−^ mice, infection results in decreased caspase-1 processing and IL-1β secretion and increased bacterial burdens [8]. Additionally, NLRC5 promotes the maturation of IL-1β mediated by NLRP3 and enhances innate immunity against LM infection by modulating neutrophil influx in vivo studies [70]. NLRC5 regulates MHC class I antigen presentation when LM infect CD8^+^ T cells, and NLRP3-mediated inflammasome activation is partly suppressed in NLRC5^−/−^ mice. Taken together, the results have shown once again that NLRC5 may be closely related to the activation of the NLRP3 inflammasome in *L. monocytogenes* infection [57].

#### 4.1.4. NLRP6 Inflammasome

NLRP6 is a novel member of the NLR family, highly expressed in the intestine and liver. Early studies generally suggested that the activation of the NLRP6 inflammasome depends on the recognition of specific pathogen-associated molecular patterns (PAMPs) [71,72].

**Figure 5:**
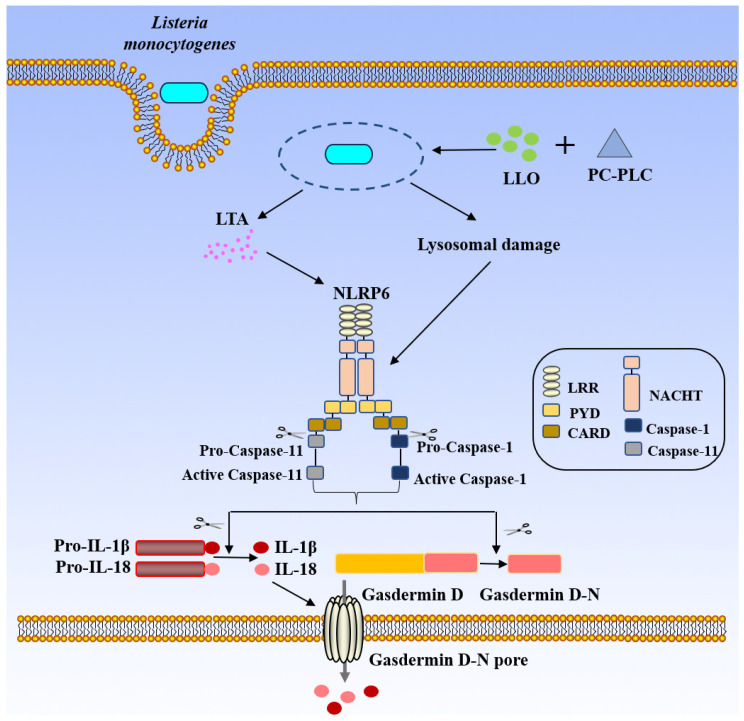
NLRP6-mediated activation of the inflammasome by *L. monocytogenes*.

Following infection of cells by *L. monocytogenes*, NLRP6 senses the released lipoteichoic acid (LTA) signal and subsequently recruits and activates both caspase-1 and caspase-11 via the adaptor protein ASC in macrophages. Although caspase-11 cannot directly process the precursors of IL-1β/IL-18, it can enhance the inflammatory response by promoting the activation of caspase-1 [7]. It is noteworthy that LTA itself possesses complex structural modifications (such as D-alanylation and glycosylation), which may influence its immunogenicity and are associated with the virulence of different bacterial strains [73,74,75]. It has been proposed that such modifications might also affect the binding efficiency of LTA to NLRP6, thereby modulating the intensity of downstream signaling; however, this has not been directly validated in the context of NLRP6 activation. A key piece of evidence from early research is that the glycerophosphate repeat backbone of LTA is essential for its ability to activate NLRP6 [37,72]. However, the most recent research has revealed a more fundamental activation mechanism, challenging the aforementioned “specific ligand” model. New evidence indicates that NLRP6 essentially functions as a general sensor for the homeostasis of the intracellular endolysosomal system. In intestinal epithelial cells, the core mechanism by which *L. monocytogenes* activates NLRP6 is not the direct binding of LTA or other PAMPs, but rather is mediated by endolysosomal damage—particularly secondary damage to double-membrane vacuoles—elicited during bacterial cytosolic survival, replication, and cell-to-cell spread. This damage generates an as-yet-undefined signal, which is sensed by the NACHT domain of NLRP6. Dependent on its ATP-binding and hydrolysis activity, this sensing drives receptor oligomerization, leading to the assembly of the canonical NLRP6–ASC–caspase-1 inflammasome and ultimately resulting in GSDMD-dependent pyroptosis and IL-1β release [76] (Figure 5).

Currently, there are two distinct models regarding the activation mechanism of NLRP6: one is the traditional model of specific PAMP recognition (e.g., LTA), and the other is the newly proposed model of endolysosomal damage sensing. This divergence in understanding may stem from differences in cell types, experimental systems, or stages of pathogen infection. Future research urgently needs to integrate these two perspectives, focusing on: (1) validating the new mechanism in physiological, endogenously expressing systems; (2) identifying the specific molecular signal directly sensed by NLRP6 during endolysosomal damage; (3) clarifying under which pathophysiological contexts one mechanism predominates over the other. Addressing these questions will be crucial for understanding the complex functions of NLRP6 in infection, inflammation, and related diseases.

Studies have shown that NLRP6^−/−^ mice infected with *L. monocytogenes* via oral gavage are largely protected from bacterial infection and exhibit lower bacterial loads in the spleen and liver, with a survival rate of 75% [8]. Overall, these findings indicate that NLRP6 plays a crucial role in *L. monocytogenes* infection.

NLRP6 recognizes LTA (lipoteichoic acid) releases from *L. monocytogenes*, and the NLRP6 inflammasome induces the processing of caspase-11 and caspase-1, which promotes the production of activated caspase-1. The activated caspase-1 cleaves Gasdermin D to produce active Gasdermin D-N, which causes the production and release of IL-1β and IL-18 and cell pyroptosis. Additionally, in intestinal epithelial cells, *L. monocytogenes* induces endolysosomal damage, which generates a signal that is sensed by the NACHT domain of NLRP6; this triggers ATP-dependent NLRP6 oligomerization and canonical inflammasome assembly, culminating in Gasdermin D-mediated pyroptosis and IL-1β release. (LTA: lipoteichoic acid; Pro-IL-1β: Pro-Interleukin-1 beta; Pro-IL-18: Pro-Interleukin-18; IL-1β: Interleukin-1 beta; IL-18: Interleukin-18; LRR: Leucine-Rich Repeat(s); PYD: Pyrin domain; CARD: Caspase Activation and Recruitment Domain; NACHT/NOD: Nucleotide-binding and Oligomerization Domain; Caspase-1: Cysteine-dependent aspartate-directed protease-1; Caspase-11: Cysteine-dependent aspartate-directed protease 11; NLRP6: NOD-like receptor family, pyrin domain containing 6).

#### 4.1.5. NLRP1 Inflammasome

NLRP1 is a NOD-like receptor that oligomerizes to form a pro-caspase-1 activation plat form for inflammasome assembly [77]. The NLRP1 inflammasome cannot be activated without self-cleavage of the FIND domain [32], whereas disrupting the FIND domain blocks the activation of the NLRP1 inflammasome [78]. The lethal factor of anthrax toxin (LeTx) can activate NLRP1 owing to the self-hydrolysis of LeTx’s lethal factor (LF) protease subunit, which can activate NLRP1 via the N-terminal-mediated proteasome degradation pathway, completing inflammasome assembly and downstream activation [33,79]. Moreover, NLRP1 is activated when cells are deprived of glucose or treated with metabolic inhibitors [34]. The specific reason may be related to ATP levels linked to inflammasome activation; hypoxia and glucose starvation cause ATP levels to decrease, which in turn triggers the activation of the mouse NLRP1 inflammasome [35].

**Figure 6:**
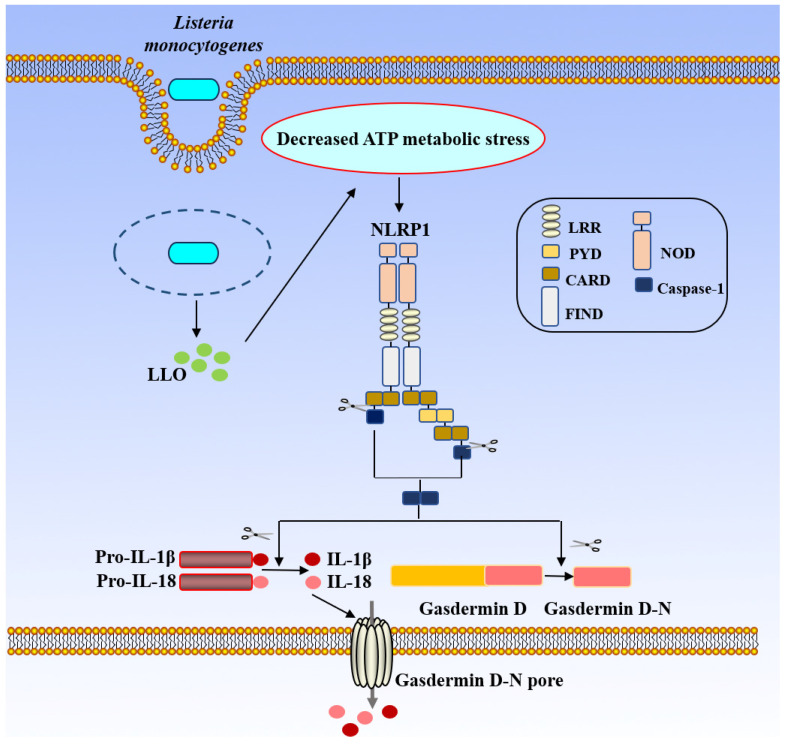
NLRP1-mediated activation of the inflammasome by *L. monocytogenes*.

*L. monocytogenes* activates the NLRP1 inflammasome by causing metabolic stress depend on the expression of LLO, which decreases the amount of cytosolic ATP [7]. Additionally, since mutations in the FIND domain prevent inflammasome activation, the N-terminal region of NLRP1 is necessary for the activation caused by *L. monocytogenes* (Figure 6) [80]. Furthermore, *L. monocytogenes*-induced activation of NLRP1 plays a role in the pathogen-induced immune response [81].

*L. monocytogenes* releases LLO and activates the NLRP1 inflammasome by reducing ATP levels. The NLRP1 inflammasome activated by metabolic stress induced *L. monocytogenes* infection, resulting in the production of IL-1β and IL-18 and mounting an antibacterial response. Furthermore, FIND domain plays a key role in the activation of NLRP1 inflammasome. (LLO: Listeriolysin O; Pro-IL-1β: Pro-Interleukin-1 beta; Pro-IL-18: Pro-Interleukin-18; IL-1β: Interleukin-1 beta; IL-18: Interleukin-18; LRR: Leucine-Rich Repeat(s); PYD: Pyrin domain; CARD: Caspase Activation and Recruitment Domain; NOD: Nucleotide-binding and Oligomerization Domain; FIND: Function to Find domain; Caspase-1: Cysteine-dependent aspartate-directed protease-1; NLRP1: NLR family pyrin domain containing 1).

#### 4.1.6. NLRC2 Inflammasome

**Figure 7:**
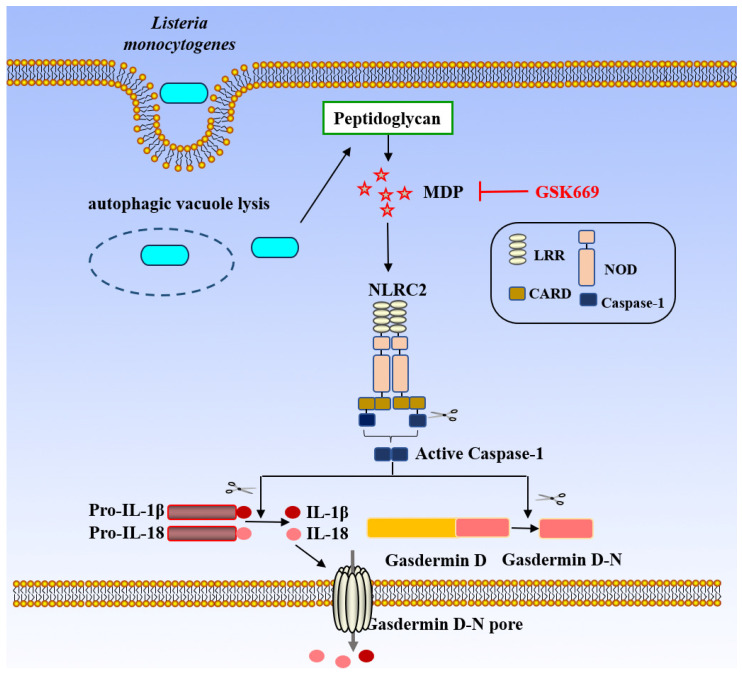
NLRC2-mediated activation of the inflammasome by *L. monocytogenes*.

NLRC2 in the cytoplasm recognizes the degradation product of *L. monocytogenes* peptidoglycan, MDP, and results in the production of antimicrobial peptides and proinflammatory factors, which depends on NF-κB, p38 mitogen-activated protein kinase, and receptor-interacting protein 2 (Rip-2) (Figure 7). As a result, mice deficient in Rip-2 are more vulnerable to infection by *L. monocytogenes* [57]. Additionally, research has demonstrated that NLRC2^−/−^ mice are more susceptible to infection than wild-type mice by oral gavage inoculation of *L. monocytogenes*. Furthermore, bacterial clearance compromised, and survival rates decrease in NLRC2^−/−^ mice treated with medicine, suggesting a critical function for NLRC2 in the pathogenic process of *L. monocytogenes* [82].

NLRC2 in the cytoplasm recognize muramyl dipeptide (MDP), the degradation product of *L. monocytogenes* peptidoglycan, NLRC2 inflammasome activation produces active caspase-1, and promotes release of IL-1β and IL-18 and cell pyroptosis. The addition of the inhibitor GSK669 suppresses MDP-induced stimulation, thereby inhibiting NLRC2 activation. (MDP: muramyl dipeptide; Pro-IL-1β: Pro-Interleukin-1 beta; Pro-IL-18: Pro-Interleukin-18; IL-1β: Interleukin-1 beta; IL-18: Interleukin-18; LRR: Leucine-Rich Repeat(s); CARD: Caspase Activation and Recruitment Domain; NOD: Nucleotide-binding and Oligomerization Domain; Caspase-1: Cysteine-dependent aspartate-directed protease-1; NLRC2: NOD-like receptor family, CARD domain containing 2).

### 4.2. AIM2 Inflammasome

**Figure 8:**
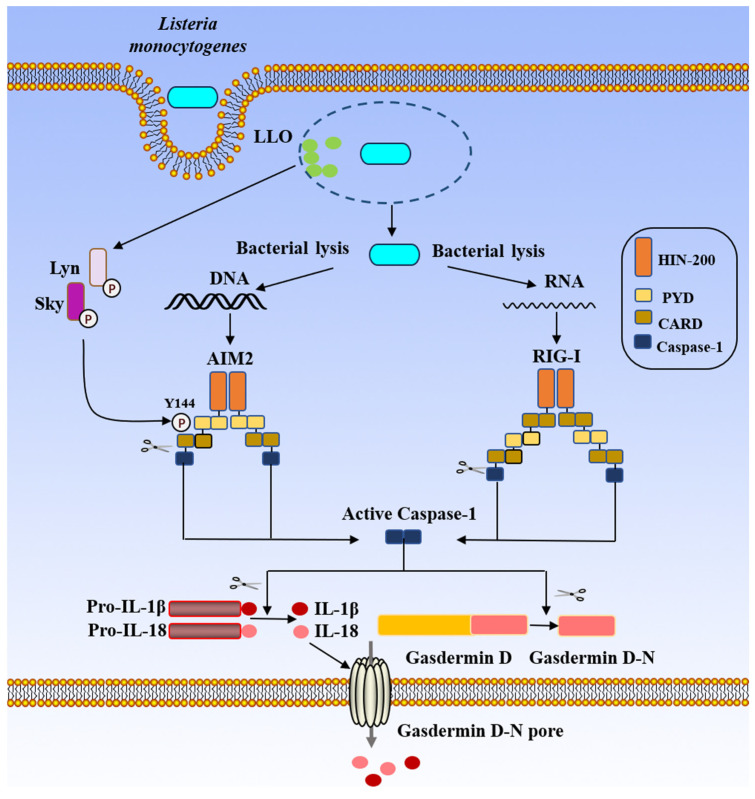
AIM2 and RIG-I inflammasomes activation in *L. monocytogenes*.

AIM2 belongs to the PYHIN family and is composed of two structural domains: the N-terminal pyrin domain and the C-terminal HIN-200 domain. AIM2 attaches to double-stranded DNA (dsDNA) via its C-terminal HIN domain, which subsequently releases the N-terminal PYD domain and interacts with ASC. ASC then recruits procaspase-1, which forms the AIM2 inflammasome. The activation mechanism of AIM2 is a protein that senses dsDNA in the cytoplasm, regardless of the source—bacteria, viruses, or host cells [39,40,41]. Double-stranded DNA stimulation of macrophages can greatly increase the level of intracellular AIM2, which in turn can cause an inflammatory response [83]. DNA not only activates the AIM2 inflammasome but also induces type I interferon (IFN) through the cGAS-STING pathway, mediating the transcriptional upregulation of AIM2 [84,85]. In THP-1β human monocytes, RNA-mediated interference inhibits the production of IL-1β elicited by DNA, resulting in a decrease in AIM2 expression, which suggests that endogenous AIM2 is required for DNA recognition (Figure 8) [83].

The discovery that the ΔflaA mutant of *L. monocytogenes* could still cause caspase-1 activation in the absence of NLRP3 provided the initial indication that AIM2 was involved in *L. monocytogenes* infection. Furthermore, caspase-1 activation was inhibited in AIM2^−/−^ cells, suggesting that the AIM2 inflammasome could also promote caspase-1 expression [8,52]. In NLRP3^−/−^ macrophages treated with AIM2 siRNA during *L. monocytogenes* infection, the activation of caspase-1 was completely suppressed [57], providing clear evidence that *L. monocytogenes* can activate the expression of the AIM2 inflammasome. How does *L. monocytogenes* activate the AIM2 inflammasome? The co-localization of *L. monocytogenes* DNA with AIM2 and ASC specks in the cytoplasm of host cells indicates that DNA release from *L. monocytogenes* escapes into the cytoplasm of infected macrophages, triggering AIM2 oligomerization, caspase-1 activation, and pyroptosis [48,60], as well as inducing the secretion of IL-1β and IL-18 [7]. AIM2 is an interferon-inducible factor, and *L. monocytogenes* induces the expression of type I interferon in host macrophages, which may upregulate AIM2 expression following infection with this pathogen [86]. In addition to *L. monocytogenes* DNA activating AIM2, the amino acid residue threonine at position 223 in the domain 3 of LLO (LLO T223) can promote ASC phosphorylation, activating the AIM2 inflammasome through Lyn-Syk signaling and interactions with membrane rafts, with the phosphorylation of ASC at the Y144 site being crucial for AIM2 inflammasome activation [58].

### 4.3. RIG-I Inflammasome

RIG-I is a cytoplasmic pattern recognition receptor with two CARD domains, an RNA helicase domain and a C-terminal domain [87]. RIG-I serves as a sensor for RNA, detecting double-stranded RNA and differentiating between self- and non-self RNA, which is crucial for antiviral immunity [42,88]. The way that double-stranded DNA activates the AIM2 inflammasome is comparable to how RIG-I activates the inflammasome. RIG-I can form a protein complex containing ASC, which can interact with other components to induce the secretion of IL-1β [89].

In mice, RIG-I expression can be upregulated in splenic reticular cells and hepatic Kupffer cells following *L. monocytogenes* infection. RAW264.7 cells express RIG-I in response to both heat-inactivated and live *L. monocytogenes*, indicating a potential function for RIG-I in the host’s innate immune response to *L. monocytogenes* infection [90]. How does the *L. monocytogenes* activate RIG-I expression? Studies have demonstrated that *L. monocytogenes* activates RIG-I by recognizing RNA produced by bacteria in the cytoplasm and that the signaling molecule CARD9 promotes the production of IL-1β (Figure 8) [57,91]. Moreover, research results imply that RIG-I sensing of *L. monocytogenes* RNA represents a non-redundant cytosolic immunorecognition route in non-immune cells without functioning in STING-dependent signaling pathway [92].

During infection, *L. monocytogenes* invades the cytoplasm through the action of LLO. LLO is crucial for the phosphorylation of the AIM2 inflammasome adaptor ASC at amino acid residue Y144 through Lyn-Syk signaling. *L. monocytogenes* releases double-stranded DNA induces AIM2 inflammasome activation via HIN-200 domain. *L. monocytogenes* secreted single-stranded RNA caused RIG-I-dependent IL-1β production and inflammasome activation via signaling molecule CARD9 domain. (LLO: Listeriolysin O; Lyn: Lyn proto-oncogene, Src family tyrosine kinase; Syk: spleen tyrosine kinase; Pro-IL-1β: Pro-Interleukin-1 beta; Pro-IL-18: Pro-Interleukin-18; IL-1β: Interleukin-1 beta; IL-18: Interleukin-18; HIN-200: Hematopoietic Interferon-inducible Nuclear proteins with a 200-amino-acid repeat; PYD: Pyrin domain; CARD: Caspase Activation and Recruitment Domain; Caspase-1: Cysteine-dependent aspartate-directed protease-1; AIM2: Absent in melanoma 2; RIG-1: Retinoic acid-inducible gene-1).

## 5. Some Promising Inflammasome Inhibitors for Inflammatory Diseases

Inflammasomes are activated in response to dangerous stimuli, leading to caspase-1 activation, proinflammatory cytokine release, and pyroptotic cell death, thereby playing a critical role in the pathogenesis of inflammatory diseases. Accordingly, inflammasome inhibitor therapy has a therapeutic benefit in these diseases. To date, many inhibitors can target different structural sites of the inflammasome, such as ASC inhibitors, caspase inhibitors, GSDMD inhibitors, anti-IL-8, IL-1β inhibitors, etc., which are related to the structure of the inflammasome (Table 2). Among them, biological agents targeting IL-1β are currently the only drugs approved for clinical use as inflammasome blockers [93].

The development of targeted medicines that act on NLRP3 or other component proteins is an inflammasome inhibitor due to the thorough investigation of the activation mechanisms of the NLRP3 inflammasome. Currently, several NLRP3 inflammasome inhibitors, including MCC950, OLT1177, oridonin, CY-09, and tranilast, have shown promise as treatment agents for a range of illnesses. MCC950 has been widely investigated, especially in relation to disorders related to NLRP3. *L. monocytogenes* induces inflammatory responses in the brains of mice through NLRP3 inflammasome activation; treatment with MCC950 in infected mice leads to the normalization of neuronal morphology and reduced inflammatory responses [51]. The function of MCC950 implies that NLRP3 may be a viable alternative target for the management of listeriosis (seen in Table 3). Additionally, in murine S. suis-infection model, MCC950 reduced the bacterial load and the excessive inflammatory response in mice brains [94].

OLT1177, a novel NLRP3 inhibitor, has completed Phase II clinical trials for the treatment of arthritis, acute gout attacks, and heart failure [95]. It has also been shown to inhibit the neutrophil NETosis response induced by the dengue virus envelope protein domain III (EIII) [96]. Oridonin has therapeutic effects on peritonitis, gouty arthritis, and type 2 diabetes and infectious diseases [97]. Tranilast reduces intestinal barrier and visceral pain by blocking the NLRP3 inflammasome in a rat model of irritable bowel syndrome (IBS) [98]. Britannin is a new NLRP3 inhibitor that inhibits human and murine macrophages from undergoing NLRP3-mediated cell pyroptosis [99].

In addition to the widely used NLRP3 inhibitors, other inflammasome inhibitors are being discovered and utilized. Among the identified NLRC4 inhibitors, which include genipin and the novel compound CS 82, genipin notably confers protective effects against pulmonary inflammation [100]. NLRC2 inhibitors such as aryl-Cr(CO)_3_ and GSK669 can treat serious inflammatory diseases associated with NLRC2 dysfunction, such as Crohn’s disease (CD), Blau syndrome (BS), and early-onset sarcoidosis (EOS) [101,102]. AIM2 inhibitors such as quercetin and andrographolide exhibit notable anti-inflammatory properties. Quercetin has been shown to mitigate inflammatory responses associated with skin diseases [103], while andrographolide significantly ameliorates radiation-induced lung injury, reducing the incidence of radiation pneumonitis and pulmonary fibrosis [104]. In conclusion, inflammasome inhibitors represent a significant frontier in immunology and drug development, owing to their broad therapeutic potential in modulating inflammatory responses, treating inflammatory diseases, and enabling novel treatment strategies.

**Table 2:** Some pharmacological inhibitors of inflammasomes.

Name of Inflammasomes	Inhibitor	Inhibition Mechanism	References
NLRP3	MCC950	Inhibition of ASC oligomerization	[97]
	CY-09	Inhibition of ATPase activity	[105]
	OLT1177	Inhibition of ATPase activity	[106]
	Oridonin	Blocking the interaction between NLRP3 and NEK7	[107]
	Tranilast	Blocking the oligomerization of NLRP3	[108]
	Licochalcone B	Interfering with the interaction between NLRP3 and NEK7	[109]
	BHB	Blocking potassium efflux and ASC oligomerization	[95]
NLRC2	Aromatic hydrocarbons-Cr(CO)_3_	Inhibition of the NF-kB signaling pathway	[101]
	GSK669	Inhibition of MDP stimulation	[102]
NLRC4	Genipin	Inhibition of autophagy	[100]
AIM2	RGFP966	Promotion of the acetylation of STAT1	[110]
	Andrographolide	Preventing AIM2 from translocating to the nucleus	[104]

**Table 3:** Examples of inflammasome inhibitors used in *L. monocytogenes* infection.

Experimental Model	Inhibitor	Inhibitor Concentration	Dosing Frequency of the Inhibitor	Results	References
BALB/c mice	Baicalin (BA)	BA-L (1 mg/100 g) BA-M (2 mg/100 g) BA-H (4 mg/100 g)	By oral gavage for six consecutive days	The BA (medium and high doses) treatment groups exhibited significantly lower embryo loss rates compared to the *L. monocytogenes* infection group through the JNK/NEK7-NLRP3/GSDMD pathway	[111]
Female C57BL/6 mice	MCC950	10 mg/kg	Single intravenous injection	Treatment with MCC-950 promoted a trend toward normalized neuronal morphology in the mouse central nervous system (CNS) relative to the *L. monocytogenes* infection group.	[51]
Mouse microglial cell line BV2	MCC950	20 μM	Pretreatment for 1 h prior to infection	MCC950 alleviates *L. monocytogenes*-induced pyroptosis in BV2 cells by inhibiting NLRP3 inflammasome activation.	[51]
HCMEC/D3 human cerebral microvascular endothelial cells	MCC950	2.5 μM	Pretreatment for 2 h prior to infection	Through suppression of the NLRP3/Caspase-1/IL-1β pathway, MCC950 alleviated the *L. monocytogenes* infection-induced downregulation of tight junction proteins in cerebral microvascular endothelial cells.	[112]

## 6. Conclusions

*L. monocytogenes* is a widely distributed zoonotic pathogen capable of causing severe infections such as encephalitis, meningitis, sepsis, abscesses, and miscarriage. Concurrently, prolonged or high-dose antibiotic use readily induces drug resistance, posing a serious challenge to clinical prevention and treatment. Therefore, there is an urgent need to develop novel adjuvant therapeutic strategies for the effective control and management of listeriosis. Within the host innate immune system, the inflammasome plays a critical defensive role by recognizing virulence factors and nucleic acid components released by the bacteria during infection. Integrating the latest research advances, this review systematically summarizes the molecular mechanisms by which *L. monocytogenes* activates various inflammasomes, with a focus on elucidating their role in the pathogenic process. It further confirms that modulating inflammasome activity with inhibitors can significantly alleviate infection-induced pathological damage. In conclusion, inflammasome activation is crucial in the pathogenic mechanism of *L. monocytogenes*. Delineating its activation pathways and targeting this signaling axis not only provides a theoretical basis for mitigating associated inflammatory injury but also points to new directions for developing more effective intervention strategies.

## 7. Future Perspectives

In the future, in-depth exploration of the association between inflammasome inhibitors and disease targets will provide important ideas for the treatment of inflammation related diseases. For interventions targeting the inflammasome pathway, it is necessary to distinguish between “therapeutic inhibition” and “preventive immune enhancement”. At the therapeutic level, inflammasome inhibitors mainly target the high inflammatory infection stage, such as MCC950, which can effectively regulate the excessive inflammatory response caused by *Listeria monocytogenes* infection, thereby reducing tissue damage. At the prevention level, vaccine development is closely related to host immune regulation. We can use the activation mechanism of inflammasomes to explore more effective vaccine adjuvants and enhance the body’s immune response against Listeria infection. 

Unlike inhibitors, the core of vaccine development lies in acquired immunity, which establishes immune memory by inducing antigen-specific T cell or antibody responses, thereby clearing pathogens faster and stronger upon exposure or reinfection. As a key innate immune hub, inflammasomes trigger cytokine responses that regulate adaptive immunity, specifically by affecting antigen presentation and T cell subpopulation differentiation. Recent studies have reported a novel inflammasome-activating nanovaccine platform (PAI), which can self-assemble into stable nanoparticles and enhance dendritic cell antigen processing as well as antigen-specific CD8+ T-cell responses through localized activation of the NLRP3 inflammasome. Animal studies further demonstrated that PAI significantly improves the antitumor efficacy of neoantigen vaccines while reducing systemic inflammation and off-target toxicity, highlighting its promising translational potential [113]. In addition, vaccine adjuvants can enhance immunogenicity through activation of the NLRP3 inflammasome, whose key role is to bridge innate and adaptive immunity. Specifically, activation of the NLRP3 inflammasome promotes the maturation of antigen-presenting cells (APCs), enabling more efficient loading of antigens onto major histocompatibility complex (MHC) molecules and upregulation of the costimulatory molecules CD80 and CD86. Meanwhile, dendritic cells increase the production and release of stimulatory cytokines such as IL-12, thereby promoting the polarization of naïve T cells and their subsequent differentiation into effector and memory T cells, ultimately initiating T cell-mediated adaptive immune responses [114]. Therefore, in the future application of inflammasomes in vaccine development, their activity should not be simply simulated, but their related innate immune signals should be used as clues for adjuvant design. On the basis of ensuring safety, the immune effect of antigens should be enhanced and the immune response optimized.

At the mechanistic level, in addition to the known inflammasome receptors NLRP1, NLRC2, NLRP3, NLRC4, and NLRP6, some new NLR members have been discovered in recent years. For example, studies have shown that NLRP9 may play a role in infection and inflammation; although the function of NLRP12 is not completely clear, there is recent evidence that it may be involved in the identification of Yersinia pestis. However, it is currently unclear whether infection with *L. monocytogenes* can activate NLRP9 or other less studied NLRs. It is worth noting that *L. monocytogenes* infection does not simply activate a certain inflammasome, but may induce complex “dialogue” mechanisms between multiple inflammasomes. However, further research is needed on how different types of inflammasomes collaborate or antagonize each other, as well as the specific signaling pathways involved. Therefore, how *L. monocytogenes* regulates the network mechanisms of various inflammasomes will be the focus of future research and will help us to better understand its pathogenic process. In summary, further clarifying the intricate interactions between *L. monocytogenes* and inflammasomes can not only provide new clues for the development of vaccines and drugs for *L. monocytogenes*, but also lay an important foundation for the prevention and control strategies of other infectious or inflammatory diseases.

## Figures and Tables

**Table 1:** Classification of Common Inflammasomes and Activation Mechanisms.

Classification of Inflammasomes	Name of Inflammasomes	Activation Mechanism	References
NOD-like receptors (NLRs)	The typical activation pathway of the NLRP3 inflammasomes	Ion flux	[26,27]
		Lysosomal rupture	[27]
		Inhibition of ROS activity	[28]
		Mitochondrial release of DNA	[27]
		NEK7	[29]
	Atypical NLRP3 inflammasome activation pathway	LPS	[30]
	NLRC4 inflammasome	Flagellin protein	[31]
		T3SS rod protein	[31]
		T3SS needle protein	[31]
	NLRP1 inflammasome	FIND self-cleavage	[32]
		Anthrax toxin lethal factor	[33]
		Metabolic inhibitor	[34]
		Decrease in ATP level	[35]
	NLRC5 inflammasome NLRP6 inflammasome	LPS	[36]
		LTA	[37]
		LPS	[38]
AIM2-like receptors	AIM2 inflammasome	Double-stranded DNA	[39,40,41]
RIG-I-like receptors	RIG-I inflammasome	RNA	[42]

## Data Availability

No data were created or analysed during this study. Data sharing is not applicable.

## Abbreviations

The following abbreviations are used in this manuscript:

PRRs	Pattern recognition receptors
LM	*L. monocytogenes*
LLO	listeriolysin O
PlcA	Phospholipase A
SLAPs	Spacious *Listeria*-containing phagosomes
NLRs	NOD-like Receptors
AIM2	Absent in melanoma 2
RIG-1	Retinoic acid-inducible gene-1
C-Met	Hepatocyte growth factor receptor
PAMPs	Pathogen-associated molecular patterns
DAMPs	Damage-associated molecular patterns
LPSs	Lipopolysaccharides
PBMCs	Peripheral blood monolayer cells
IRAK1	Interleukin-1 receptor-associated kinase 1
TLRs	Toll-like receptors
NF-κB	Nuclear factor-κB
NAIPs	NLR family apoptosis-inhibiting proteins
T3SS	Type III secretion system
LTA	Lipoteichoic acid
Rip-2	Receptor-interacting protein 2
MDP	Muramyl dipeptide
